# Balloon cells in malformations of cortical development: friends or foes?

**DOI:** 10.1186/s42494-024-00164-5

**Published:** 2024-06-14

**Authors:** Zili Liu, Xuefeng Shen, Kaomin Lin, Fengpeng Wang, Jin Gao, Yi Yao, Jianyuan Sun

**Affiliations:** 1 CAS Key Laboratory of Brain Connectome and Manipulation, Shenzhen-Hong Kong Institute of Brain Science, Shenzhen Institute of Advanced Technology (SIAT), Chinese Academy of Sciences, Shenzhen, 518055 China; 2 The Brain Cognition and Brain Disease Institute, SIAT, CAS, Shenzhen, 518055 China; 3 Epilepsy Center, Fujian Medical University Affiliated Xiamen Humanity Hospital, Xiamen, 361003 China; 4 Department of Pathology, Fujian Medical University Affiliated Xiamen Humanity Hospital, Xiamen, 361003 China; 5 HH-SIAT Joint Center for Epilepsy Research, Fujian Medical University Affiliated Xiamen Humanity Hospital, Xiamen, 361003 China

**Keywords:** Balloon cells, MCD, Epilepsy, mTOR pathway

## Abstract

Balloon cells (BCs) are specific pathological marker of cortical malformations during brain development, often associated with epilepsy and development delay. Although a large number of studies have investigated the role of BCs in these diseases, the specific function of BCs as either epileptogenic or antiepileptic remains controversial. Therefore, we reviewed literatures on BCs, delved into the molecular mechanisms and signaling pathways, and updated their profile in several aspects. Firstly, BCs are heterogeneous and some of them show progenitor/stem cell characteristics. Secondly, BCs are relatively silent in electrophysiology but not completely isolated from their surroundings. Notably, abnormal mTOR signaling and aberrant immunogenic process have been observed within BCs-containing malformations of cortical development (MCDs). The question whether BCs function as the evildoer or the defender in BCs-containing MCDs is further discussed. Importantly, this review provides perspectives on future investigations of the potential role of BCs in epilepsy.

## Introduction

 Malformations of cerebral cortical development (MCDs) include a wide range of developmental disorders that are common causes of epilepsy or/and developmental delay [1, 2]. MCDs can be classified into different subtypes based on their clinicopathological features. Balloon cell is a histopathological hallmark frequently observed in the lesion areas of several MCD subtypes, such as focal cortical dysplasia type IIb (FCD IIb), tuberous sclerosis complex (TSC), and hemimegaloencephaly (HME) [3–7].

Prior researches have extensively investigated BCs and provided valuable insights into their morphology, distribution, genetic mutations, transcriptomic patterns, protein expressions, electrophysiological properties, and signaling pathways [5, 8–10]. These studies have contributed to our initial understanding of BCs and shed light on the potential pathomechanisms underlying MCDs and drug-resistant epilepsies. However, some of the findings have resulted in conflicting conclusions, and the exact epileptogenic mechanisms are still not completely comprehended, calling for further investigations to faithfully uncover the precise role of BCs [11].

In this review, we aim to provide a comprehensive summary of the current literature on BCs. Beginning with an overview of several BCs-containing MCDs, we further summarize the features of BCs, including their origin, cell cycle regulation, electrophysiology, etc. Moreover, we delve into the molecular mechanisms and signaling pathways related to BCs, which help to understand the evildoer or defender role of BCs in these BCs-containing MCDs. A better understanding of BCs would aid in comprehending the pathogenesis of epilepsy and lead to more effective therapeutic strategies.

## Balloon cells-containing malformations of cortical development

MCDs are characterized by abnormal cortical structure or presence of heterotopic grey matter, sometimes associated with abnormal brain volume [1]. MCDs may cause severe morbidity at any age, but common symptom onset ranges from early childhood to early adult age [2]. Approximately 40–50% of drug-resistant epilepsies treated with surgery in children are caused by MCDs [1].

**Fig. 1:**
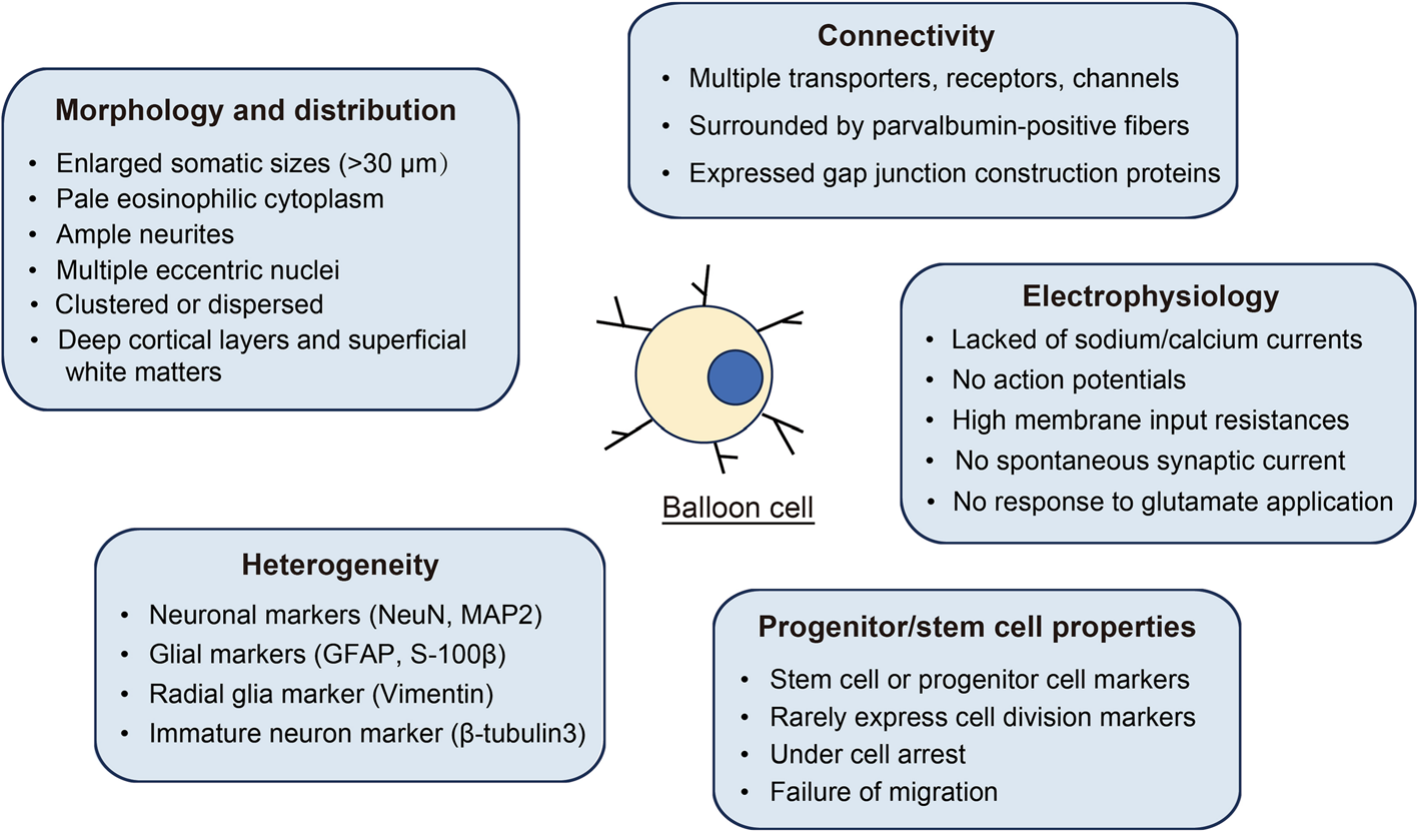
An overview of the features of BCs

MCDs are typically classified based on neuroimaging features, clinical phenotypes and genetic findings [2]. In surgically resected tissues, BCs are characterized by their enlarged somatic sizes, pale glassy eosinophilic cytoplasm in H&E staining, multiple eccentric nuclei, and ample neurites but minimal axonal processes (Fig. 1). Notably, they lack detectable Nissl bodies, distinguishing them from other neurons [9, 12–14]. Therefore, even though the classification schemes of MCDs change constantly, BCs-containing MCDs remain widely accepted [1, 2, 15–17].

One typical BCs-containing MCDs subtype is FCD type IIb [2, 10, 18, 19]. FCD was first identified by Taylor and colleagues in 1971, and was manifested as localized malformation and abnormal development of the cortex [20, 21]. FCDs were categorized into three groups: FCD I, characterized by alterations in columnar/radial (Ia) or laminar/tangential structure (Ib); FCD II, often easily visualized by MRI and characterized by marked disruption of cortical lamination with presence of morphologically abnormal cell types, specifically dysmorphic neurons (IIa) and ones with balloon cells (IIb); and FCD III, associated with additional brain lesions in the same lobe, such as hippocampal sclerosis (IIIa), tumor (IIIb), vascular malformation (IIIc), or lesions acquired during early life (IIId) [1, 6, 7, 18, 22, 23].

Seizures are better controlled in FCD II than in FCD I, despite the presence of more severe histopathologic lesions in FCD II, such as misplaced cytomegalic dysmorphic neurons, reduced white matter myelin content, and blurring of the white/grey matter boundary [22, 24]. BCs are frequently found in clusters or dispersed throughout FCD IIb lesions, with a preference for localizing in deep cortical layers and superficial white matter [22].

Another common subtype of BCs-containing MCDs is TSC, which is an autosomal dominant, multi-system disorder resulting from mutations in the *TSC1* or *TSC2* genes [4, 20, 25, 26]. Over 80% of TSC patients develop cortical tubers, which are focal malformations that form during brain development [27]. Like FCD IIb, cortical tubers in TSC also exhibit laminar disorganization and the presence of BCs [28, 29].

Another type of BCs-containing MCDs is HME, which is a congenital brain malformation that mainly affects one cerebral hemisphere and sometimes involving the ipsilateral cerebellar hemisphere and brainstem [30, 31]. The histopathological features of HME encompass abnormalities in cellular growth and cytomorphology, such as the presence of BCs in both gray matter and superficial white matter, as well as disorganized tissue architecture [31, 32].

BCs have been identified as a specific feature of FCD IIb, TSC and HME and are linked to severe brain disorders and drug-resistant epilepsies. Despite progress in the classification and characterization of BCs, their cellular origin and functional implications in MCDs remain unclear.

## An origin profile of balloon cells: heterogeneity and cell cycle arrest

**Fig. 2:**
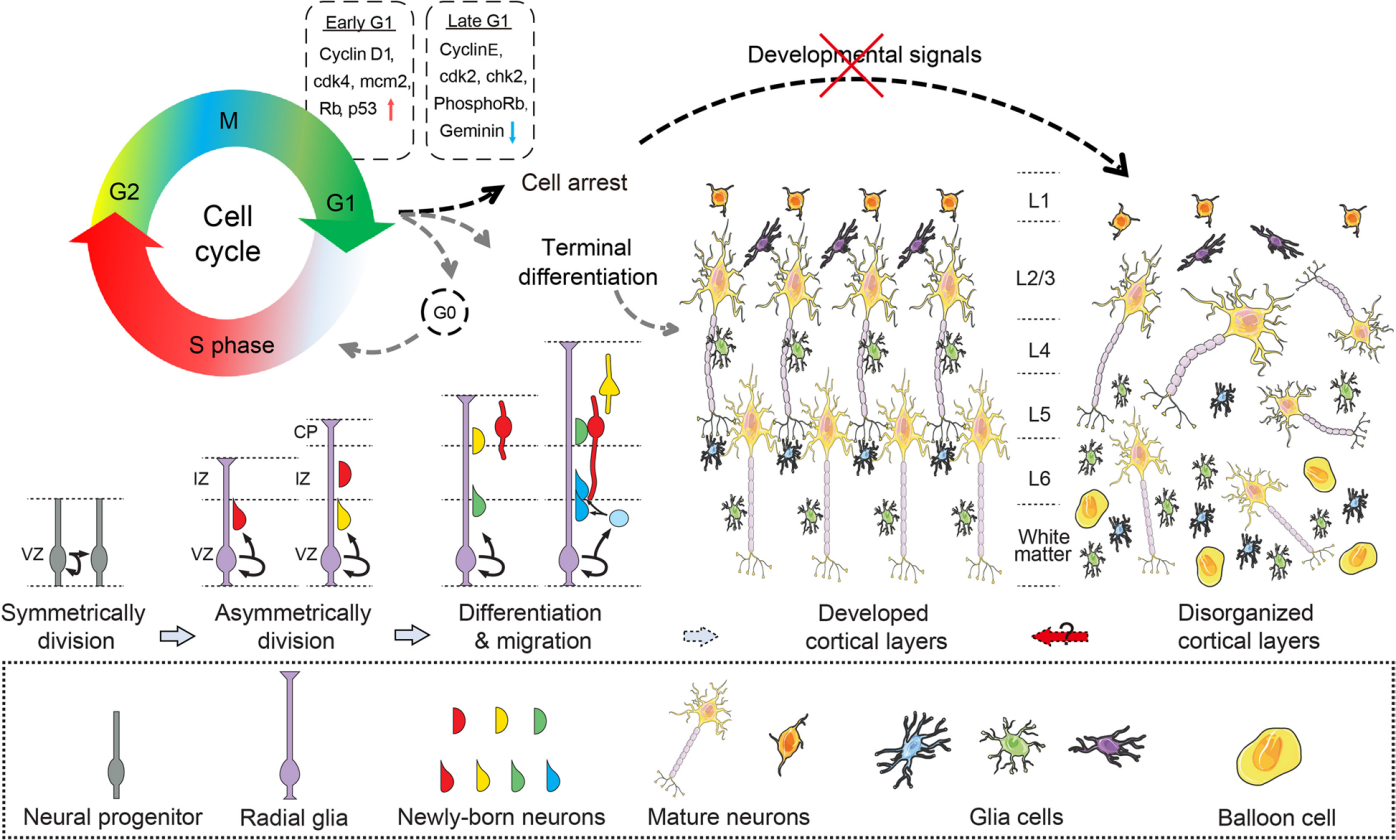
The schematic depicts the cell cycle, differentiation and regulation of cortical development. Adapted from Dalton et al., Thom et al., and Luo et al. [40–42]. In the early stage of development, neuroepithelial cells or neural progenitors in the ventricular zone (VZ) divide symmetrically. As development progresses, they transform into radial glia and extend their processes to intermediate zone (IZ) and cortical plate (CP). The neurons generated from radial glia through asymmetric division initiate migration along the radial glia and settle in the deepest layer. Subsequently, new-born neurons migrate past the early-born neurons to settle in progressively more superficial layers.  In the top-left corner, a cell cycle is illustrated by a gradient color ring, demonstrating the four primary cell cycle stages. Stem cells are refractory to differentiation signals during S, G2 and M phases. Upon entry into G1 phase, cells become permissive for cell fate specification and responsive to developmental signals. The G1 phase can be further divided into early G1 and late G1, expressing specific regulatory proteins listed in the dashed boxes respectively. BCs expressed abundant early G1 proteins and few late G1 proteins, indicating that BCs are trapped in early G1 phase. Dysregulation of cell cycle proteins in BCs may be a primary abnormality that affects cell maturity, cell cycle progression and the determination of cell fate. This dysregulation is responsible for the disorganized cortical layers in FCDs and some other pathological processes

The central nervous system (CNS) is one of the first organ systems to initiate development in the human body, exhibiting rapid growth from 4 postconceptional weeks (PCWs) to the third postnatal year [33]. This development process intricately involves the interplay of morphogens and transcription factor gradients, acting on various cortical progenitor cells [34]. The evolution of the human brain has been systematically delineated by several groups [33]. Specifically, neuroepithelial cells in the ventricular zone (VZ) act as the stem or progenitor cells for all neurons and macroglia in the CNS. These cells undergo sysmmetrical divisions to expand their populations during the early embryonic stages [35]. Starting at 7 PCWs, neural progenitor cells transition into radial glia (RG), extending long processes from the ventricular to the pial surface. Subsequently, they undergo asymmetric division, yielding one RG and one post-mitotic neuron or an intermediate progenitor cell [36–38]. Newly-generated neurons migrate in an “inside out” pattern, moving past the early-born neurons to form progressively superficial layers. In humans, this migration is projected to last for 143 days (48–191 postconceptional days), compared to 11 days in mice and 67 days in rhesus macaques (Fig. 2) [33–35, 37–39]. Dysregulation of these developmental processes can impact the structure and functionality of the CNS, potentially leading to neurological or psychiatric disorders.

To date, there is scant evidence indicating the presence of BCs in either the typical human cerebral cortex or any animal model, suggesting that they might represent a unique cell type specifically linked to the development of MCDs, or they may be a modified version of an existing cell type with abnormal morphology. To fully understand the mechanism underlying the origin of BCs, it is necessary to identify their developmental characteristics.

BCs express a heterogeneous and frequently mixed array of lineage markers [3, 5, 7, 10, 11, 14, 30]. These markers include both neuronal and glial markers, such as NeuN, neurofilament, MAP2, GFAP, and S-100β protein, indicating significant diversity cell types within BCs [23, 43, 44]. Vimentin, a marker typically expressed in immature or radial glia, is frequently observed in BCs as well [14, 26, 44–46]. In addition, markers such as β-tubulin 3 and TUJ1, which are commonly associated with immature neurons, have been detected in BCs [47–49].

BCs share markers with stem cells or progenitor cells, such as nestin, CD34, CD133, SOX2, BLBP, Otxl, GFAP-δ, Pax6, Klf-4, β1-integrin, and CRMP4, suggesting that BCs may derive from a lineage of neuroglial-like progenitor cells (Fig. 1) [10, 27, 44, 47, 50, 51]. Additionally, two in vitro studies demonstrated that BCs were cultured after being isolated from surgical resections of FCDs and tuber tissues, providing further validation of their stem cell characteristics [11, 27]. Despite their cellular immaturity, BCs rarely express cell division markers such as proliferating cell nuclear antigen (PCNA) or Ki-67, suggesting that these cells are in a near-stem-cell stage but are not actively dividing [30, 50, 52].

The regulation of cell proliferation, differentiation and fate commitment is tightly linked to cell cycle control signaling [40, 53]. Cells in distinct cell cycle states exhibit varied molecular features and functional outputs [54]. It is now widely accepted that the G1 phase provides a critical window for the genetic and epigenetic regulation of cell fate decisions [40, 54]. During each round of cell division, intrinsic and extrinsic factors trigger cells to decide whether to continue dividing or entering a quiescent state (G0) through a mechanism called “restriction point” (R-point) control [55].

Previous studies by Thom et al. have evaluated the proliferative potential of BCs in FCD [41, 50]. Their findings showed that the majority of BCs exhibited strong staining with the Mcm2 antibody, which is expressed throughout the G1 phase of the cell cycle [50]. Furthermore, only a small fraction of BCs expressed geminin, which is specifically expressed during S/G2/M phases, suggesting that only a few BCs entered the S phase or complete the cell cycle [41]. Based on these findings, it has been hypothesized that BCs may represent remnants of early cortical cells that have undergone cell cycle arrest and failed to undergo differentiation or to be eliminated during development (Fig. 2) [41]. Specifically, BCs may arrest in the G1/S phase transition, a stage where the cell has grown physically but before DNA replication is initiated [10, 41].

The G1 phase can be further subdivided into early and late phases, characterized by specific markers and/or regulators such as cyclin D1, cdk4, p53, and nonphosphorylated retinoblastoma protein (Rb) for the early phase, and Cyclin E, cdk2, phosphorylated Rb, and checkpoint 2 for the late phase [53]. BCs demonstrate heightened expression of regulators associated with the early G1 phase but exhibit diminished expression of regulators associated with the late G1 phase, suggesting a propensity for BCs to be trapped in the early G1 phase with limited progression into the late G1 phase (Fig. 2) [41, 52].

**Fig. 3:**
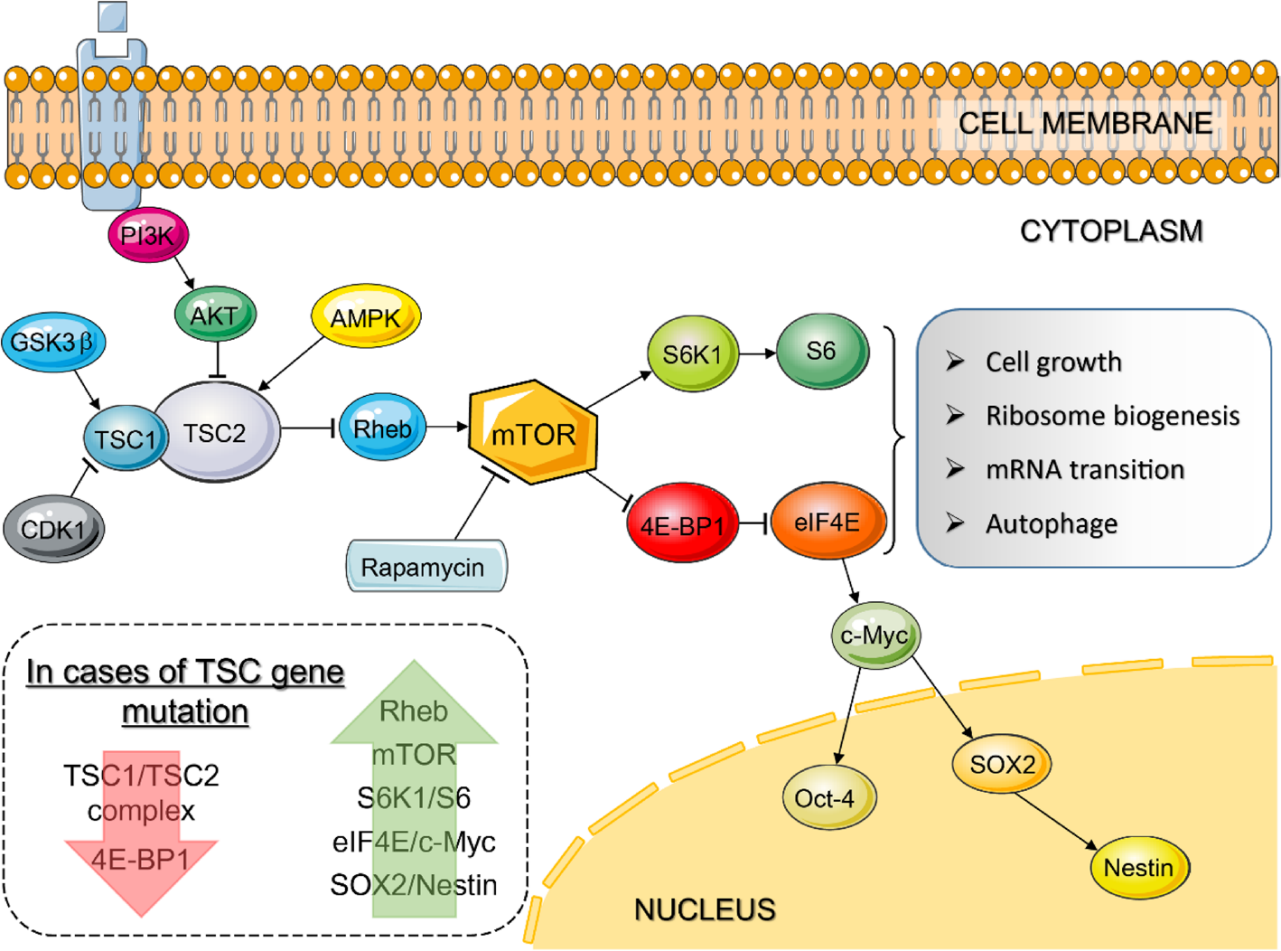
The schematic depicts the mTOR pathway. Adapted from Iffland et al. and Orlova et al. [10, 27]. The cytoplasmic mTOR signaling pathway is influenced by multiple nodes, including PI3K, AKT, AMPK, CDK1, GSK3β, etc. The TSC1/TSC2 protein complex (tuberin/hamartin complex) integrates cues from growth factors, cell cycle regulators, and nutrients to regulate the activity of mTOR signaling, which could be inhibited by rapamycin. Hyperactive mTOR signaling results in enhanced S6K1 and S6 phosphorylation, leading to increased cell size. Inhibition of 4E-BP1 by mTOR results in enhanced translation of c-Myc, which translocates into the nucleus to regulates expression of Oct-4, SOX2, and nestin, perhaps conferring the immature cellular phenotype of BCs. Constitutive activation of mTOR also enhances ribosome biogenesis, mRNA transition, protein synthesis, and inhibits autophagy. Loss-of-function mutations in either *TSC1* or *TSC2* lead to hyperactivation of the mTOR signaling pathway and elevate expressions of the downstream molecules, which might be a pathogenic mechanism in some MCD subtypes.  Abbreviation: PI3K : phosphatidylinositol 3-kinase; AMPK : AMP kinase; GSK3β : glycogen synthase kinase 3β; CDK1 : cyclin-dependent kinase 1; Rheb : Ras homologue enriched in brain; S6K1 : p70S6 kinase 1; S6 : ribosomal protein S6; 4E-BP1 : 4-elongation factor binding protein-1: eIF4E : eukaryotic initiation factor 4E; Oct-4 : octamer-4; SOX2 : sex-determining region Y-box 2

Beyond cell cycle abnormalities, the *TSC* genes in the mTOR signaling pathway have been extensively studied for their role in the pathogenesis of TSC and FCD IIb cases, particularly in BCs and dysmorphic neurons (DNs) (Fig. 3) [50, 56]. Notably, Baldassari et al. employed laser-capture microdissection (LCM) on frozen brain sections to selectively isolate BCs, DNs and morphologically normal-appearing neurons. This approach enabled a more direct genetic comparison, revealing that both BCs and DNs carry pathogenic variants related to the mTOR pathway, similar to those observed in HME cases [3]. Furthermore, they observed a significant enrichment of somatic variant in glial cells, possibly indicating an early mutational event in common neuroglial progenitors, which aligns with our previous discussion.

The tuberin/hamartin complex, which is encoded by *TSC1/TSC2*, plays a key role in the regulation of cell size, shape, proliferation, differentiation, and the cell cycle. Mutations in these genes result in the activation of the mTOR signaling pathway, which leads to increased protein synthesis, cell growth, and proliferation. Ultimately, these molecular alterations contribute to the formation of cortical dysplasia (Fig. 3) [20, 29].

Doublecortin, a fetal neuronal protein that regulates neuronal migration, is highly expressed in BCs [57, 58]. Failure of maturation and migration during development can result in the persistence of immature neurons and dysfunction of synaptic circuits, contributing to the pathogenesis of cortical dysplasia [9]. Coexpression of the anti-apoptotic protein BCL-2 with CD133-positive BCs in FCDs suggests that resistance to programmed cell death may be involved in the pathogenesis of cortical dysplasia [48].

These findings indicate that BCs are under cell cycle arrest and migration failure. It is proposed that BCs originate from naive progenitor cells whose developmental trajectory is prematurely terminated [5, 58].

## Physiology function of balloon cells: “silent” but not isolated

Dysplastic neurons (DNs) typically exhibit morphological, structural, or functional abnormalities during cortical development in the brain. They are considered important hallmark cells in the pathological diagnosis of epilepsy patients and are often observed alongside BCs [10]. Studies have reported that DNs within the lesion exhibit increased calcium influx and currents in response to stimulation, compared to normal neurons, suggesting that DNs play a significant role in generating epileptiform discharges within the MCD network [8, 13]. However, the involvement of BCs in epileptogenesis or ictal discharges has not been extensively examined in vivo, mainly due to the lack of an animal model that faithfully recapitulates BCs [8, 13, 28]. Currently,  our understanding of BCs electrophysiological activities mainly comes from ex vivo brain slice preparation derived from resected tissue. In FCD IIb- and TSC- brain slices, Mathern’s team found that BCs lacked of voltage- and ligand-gated sodium and calcium currents, did not generate action potentials when depolarized, and displayed no spontaneous synaptic currents or responses to exogenous application of glutamate [8, 9, 28]. Thus, it seems that BCs neither contribute to abnormal electrical discharges nor receive synaptic inputs [9].

However, the involvement of BCs in epileptogenesis is not completely rule out [13]. Although BCs may not generate abnormal electrical discharges spontaneously, they are not completely inactive or isolated from other cells and tissues within the lesions. This hypothesis is supported by numerous observations, including electrophysiological recordings and immunostaining of connection-related proteins [10, 59–61].

In Mathern’s electrophysiological investigations, BCs were found to exhibit an almost linear relationship in the hyperpolarizing direction but demonstrate strong rectification in the depolarizing direction, which was attributed to the activation of delayed rectifier K^+^ channels [28]. This finding is plausible because BCs express multiple transporters, receptors, which contributes to the unclear role of BCs in epileptogenesis in MCDs [9, 10, 47, 56, 59, 60, 62].

In FCD tissues, BCs express VGLUT2, a vesicular glutamate transporter that enables them to release glutamate and contribute to epileptogenesis [9, 47]. However, BCs also express EAAT2/GLT1, a glutamate transporter typically found in glia cells, indicating a potential role in glutamate buffering [9, 62]. Some studies suggest that BCs-containing areas exhibit increased clearance of glutamate, which could limit the spread of epileptic activity [9, 62]. Additionally, BCs are known to express NKCC1, a chloride transporter primarily found in immature neurons that regulates GABA_A_ receptor function by influencing the accumulation of [Cl^−^]_i_ and maintaining the Cl^−^ gradient [56]. In cortical tubers, BCs have been found to express elevated levels of LAT1, a sodium-independent transporter that facilitates the active transport of large neutral amino acids [63].

BCs express not only various transporters but also glutamate receptors subunit proteins, including AMPA receptor (GluR1-4), kainate receptor (GluR5-7), NMDA receptor (NR1, NR2a/b), and subtypes of metabotropic glutamate receptor, such as mGluR1α, mGluR2/3, mGluR5 [8, 14, 64]. BCs also exhibit immunoreactivity for proteins involved in the regulation and induction of angiogenesis during both development and in pathological conditions [65]. Additionally, there is an increased expression of ion channels on BCs, such as Panx1 and Panx2, transient receptor potential canonical channels (TRPCs), TRPV1, acid-sensing ion channels (ASICs) (Table 1) [66–73].

**Table 1:** Immunoreactivity findings of BCs in MCD specimens

Antigen/Expression		Description/Labeling	Pathology	References
**Neuropithelial**				
** Neurofilament**	+	Neuronal and axonal marker	FCD IIb, HEM	[14, 30, 64, 74]
** NeuN**	+	Neuronal nuclear protein, neuronal nuclear marker	FCD IIb, HEM	[64]
** MAP2**	+	Microtubule associated protein 2, neuronal and dendritic marker	FCD IIb, HEM	[27, 48]
** S-100β**	+	Glial marker	FCD IIb, TSC, HEM	[30, 75]
** GFAP**	+	Glial fibrillary acidic protein, astrocyte marker	FCD IIb, TSC, HEM	[14, 74]
** Chromogranin A**	+	Neuronal marker	HEM	[30]
**Progenitor/stem cell marker**				
** Vimentin**	+	Immature neurons and radial glia marker	FCD IIb, HEM	[14, 48]
** Phospho-vimentin**	+	Intermediate filament protein identified in radial glial cells	FCD IIb	[47]
** Nestin**	+	Neural stem cell marker	FCD IIb	[11, 14, 48]
** CD34**	+	Hematopoietic progenitor cells and vascular endothelium	FCD IIb	[14]
** CD133**	+	Pluripotential stem cells marker	FCD IIb, TSC	[11, 48]
** SOX2**	+	SRY-box transcription factor 2, expressed in neuroglial progenitor cells	FCD IIb, TSC	[11, 27]
** SOX3**	+	SRY-box transcription factor 3, expressed in neuroglial progenitor cells	FCD IIb	[27]
** BLBP**	+	Brain lipid binding protein, expressed in neuroglial progenitor cells and radial glial cells	FCD IIb	[47]
** Otx1**	+	Orthodenticle-1, expresed in neuroglial progenitor cells	FCD IIb	[47]
** Pax6**	+	Paired box gene 6, expressed in radial glial cells	FCD IIb	[47]
** GFAP-δ**	+	A distinct splice variant isoform of GFAP, expressed in neuronal stem cells	FCD IIb, HEM	[74, 75]
** Mcm2**	+	Minichromosome maintenance complex component 2, expressed in neural stem cells	FCD IIb	[41]
** CRMP4**	+	Collapsin response mediator protein 4, a marker for newly generated neurons	FCD IIb, TSC	[47]
** β1-integrin**	+	A stem cell marker	FCD IIb, TSC	[11]
** Klf-4**	+	Krüppel-like factor 4, a stem cell marker	FCD IIb	[27]
** Ki-67**	-	Protein phosphatase 1, expressed in neuronal precursors	FCD IIb, TSC	[41, 47]
** β-tubulin3**	+	Expressed in immature neurons	FCD IIb, TSC	[27, 48]
** TUJ1**	+	Class III β-tubulin, expressed in immature neurons	FCD IIb	[48]
**Cell cycle proteins**				
** cdk4**	+	Cyclin-dependent kinase 4	FCD IIb	[41]
** cdk2**	-	Cyclin-dependent kinase 2	FCD IIb	[41]
** p53**	+	Cellular tumor antigen p53, eell cycle protien	FCD IIb	[41]
** Rb**	+	Nonphosphorylated retinoblastoma protein, cell cycle protien	FCD IIb	[41]
** PCNA**	-	Proliferating cell nuclear antigen, a cell cycle nuclear protein	FCD IIb, TSC	[26, 47]
**Transporters**				
** VGLUT2**	+	Vesicular glutamate transporter 2	FCD IIb	[47]
** VGAT**	-	Vesicular GABA transporter	FCD IIb	[47]
** EAAT2**	+	Excitatory amino acid transporter 2, glutamate transporter of glia cells	FCD IIb	[47, 76]
** GLT1**	+	Glucose transporter type 1, a glucose transporter of glia cells	FCD IIb	[47, 76]
** NKCC1**	+	Na-K-2Cl cotransporter	FCD IIb, TSC	[56]
** KCC2**	-	K-Cl cotransporter	FCD IIb, TSC	[56]
** LAT**	+	L-type amino acid transporter	TSC	[63]
**Receptor subunits**				
** GluR1/2/3/4**	+	AMPAR subunit glutamate receptor 1/2/3/4	FCD IIb	[14]
** GluR5/6/7**	+	Kainate receptor subunit glutamate receptor 5/6/7	FCD IIb	[14]
** mGluR1α**	+	Group Iα metabotropic glutamate receptors	FCD IIb	[64]
** mGluR2/3**	+	Group II/III metabotropic glutamate receptors	FCD IIb	[64]
** mGluR5**	+	Group V metabotropic glutamate receptors	FCD IIb	[64]
** NR1**	+	NMDAR protein1	FCD IIb	[14]
** NR2a/b**	+	NMDAR protein 2a/b	FCD IIb	[14]
** RAGE**	+	Receptor for advanced glycation end products	FCD IIb	[77]
** TRPV1**	+	Transient receptor potential vanilloid receptor	FCD IIb, TSC	[66]
** CB1/2**	+	Cannabinoid receptors	FCD IIb, TSC	[78]
**Channels**				
** Panx1/2**	+	Pannexin1/2, large-pore ion channel, involved in epilepsy and brain development	FCD IIb	[70]
** TRPC1/4/6**	+	Transient receptor potential canonical channel	FCD IIb	[68, 69, 71]
** ASICs**	+	Acid-sensing ion channels, H^+^-gated cation channel	FCD IIb	[72]
** AMOG**	+	Adhesion molecule on glia, a Na^+^/K^+^-ATPase	FCD IIb	[79]
** Cx43**	+	Connexin43, a gap junction subunit	FCD IIb	[61]
**Inflammatory markers**				
** IL-6/IL-6R**	+	Cytokine interleukin 6 and its receptors	FCD IIb, TSC	[80]
** IL-17/IL-17R**	+	Cytokine interleukin 17 and its receptors	FCD IIb, TSC	[81]
** TLR2/4**	+	Toll-like receptors	FCD IIb	[77]
** HMGB1**	+	High-mobility group box 1	FCD IIb, TSC	[77]
** VEGFR-1/2/3**	+	Vascular endothelial growth factor receptors	FCD IIb	[65, 82]
** MMP9**	+	Matrix metalloproteinases 9	FCD IIb	[83]
** LILRB2**	+	Leukocyte immunoglobulin-like receptor B2	FCD IIb, TSC	[84]
** NOS**	+	Nitric oxide synthase	FCD IIb, TSC	[85]
** COX-2**	+	Cyclo-oxygenase 2	FCD IIb, TSC	[85]
**Other proteins**				
** Doublecortin**	+	A fetal neuronal protein that regulates neuronal migration	FCD IIb, TSC	[57, 74]
** DCL**	+	Doublecortin-like, regulates neuronal division and radial migration	FCD IIb, TSC	[58]
** BCL-2**	+	B-cell lymphoma-2, antiapoptotic gene products	FCD IIb	[48]
** α-B-Crystallin**	+	Marker of epileptic foci	FCD IIb, TSC	[73]

BCs show decreased parvalbumin (PV)-immunoreactivity. However, they are surrounded by abundant PV-positive fibers in the deep portions of the malformed gray matter and superficial white matter, indicating a dense cluster of GABAergic input to BCs [59, 60]. GABAergic synaptic inputs are excitatory in immature pyramidal neurons [9, 86]. In MCDs, dysplastic cells retain features of immature cortex with a predominance of GABA synaptic activity [87, 88]. It is plausible that the GABAergic input to BCs may contribute to epileptogenesis.

 In CNS, astrocytes establish interconnected networks through gap junctions (GJs) composed of connexins subtypes Cx30 and Cx43 [61, 89]. Previous studies have suggested that the prototypic form of electrically-induced seizure-like oscillations can be driven solely by fast-spiking networks through their excitatory GABAergic transmissions via gap junction-mediated communication [90, 91]. Within epileptic tissues of FCD IIb, clusters of Cx43-immunopostive puncta (but not Cx30) have been detected on subsets of BCs and astrocytes. While further validation is needed to confirm this observation and its functionality, it provides a plausible mechanism for spatial buffering of extracellular ions and neurotransmitters [61].

In sum, BCs may play a dual role in the epileptogenic network, either contributing to epileptogenesis or extering antiepileptic effect [10]. Further research is necessary to clarify the exact role of BCs in MCDs.

## Pathology pathway of balloon cells: an abnormal mTOR signaling and immunoreaction

For a long time, the pathogenesis of MCDs lacked etiological clues. In 2004, an insightful study revealed overactivation of mammalian target of rapamycin (mTOR) signaling pathway in human specimens of FCD IIb and cortical tubers obtained during epilepsy surgery [10, 92]. These studies demonstrated the presence of mTOR pathway overactivation, as evidenced by phosphorylated ribosomal S6 (PS6) and 4E-BP1 in DNs and BCs (Fig. 3) [93, 94]. In the following year, mTOR hyperactivation has been discovered in other MCD subtypes, including HME and ganglioglioma [93, 94]. Subsequent studies in cell culture and animal models replicate these observations in human specimens, further highlighting a strong association between mutations in mTOR regulatory genes and mTOR overactivation, which could be effectively blocked or reversed by mTOR inhibitors, such as rapamycin (Fig. 3) [79, 95, 96]. These findings strengthen the notion that the dysregulation of the mTOR signaling pathway plays an important role in epilepsy-associated pathologies.

The mTOR pathway is a key regulator in the development of the cerebral cortex [97, 98]. Nevertheless, it remains difficult to explain the entire pathogenesis of MCDs solely base on the mTOR pathway. For instance, mTOR-signaling hyperactivation is only present in limited cell types, like BCs and DNs in FCD and TSC specimens, leading to the hypothesis that FCDs may arise from somatic gene mutations occurring in a single or small subset of neuroglial progenitor cells in the telencephalic VZ during embryogenesis [97]. For instance, adhesion molecule on glia (AMOG), recognized as a regulator of mTOR, has been detected in reactive astrocytes, displaying robust perisomatic staining in BCs [79]. Over the past several years, both somatic and germline mutations in genes encoding mTOR-cascade regulatory proteins, including *TSC1/TSC2*, have been associated with FCD IIa and IIb [10].

Studies have shown that expressing mutant *MTOR* constructs in fetal mice brain results in aberrant cell size, neuronal migration, and cortical lamination, ultimately leading to spontaneous seizures [99]. Similarly, somatic *MTOR* mutations derived from FCD IIb patients elevate the phosphorylation level of 4E-BP1 in HEK293T cells [95]. In addition, loss-of-function mutations in *DEPDC5* have been identified in FCD IIa and IIb specimens in the forms of germline frameshift, splice-site, or nonsense variants [96, 100]. More recently, a heterozygous germline frameshift mutation in *NPRL3* was identified in FCD IIa patients through whole-exome sequencing and linkage analysis [101].

Moreover, experimental studies have shown that manipulating these gene results in altered cell morphology and hyperactive mTOR signaling. A highly consistent and reproducible feature of the tissues with abnormal mTOR pathway is the disruption of cytoarchitecture, which accounts for the enlargement of BCs and cytomegalic neurons in cortical dysplasia. This results in a focal area of the brain being abnormal while the rest of the cortex remains normal, and alters the laminar position of other normally appearing neurons [9, 92, 93, 102, 103]. In addition, mutations in mTOR pathway-related genes also lead to changes in releasable factors, neurotransmitters, and modulators, which subsequently alter cell shape and motility in adjacent seemingly unaffected cells [104–106]. It appears that at the molecular and cellular levels, FCD IIa and IIb are mTORopathies, and targeting the mTOR signaling pathway could be a potential treatment option for drug-resistant epilepsies.

Besides mTOR, the Wnt/Notch pathway, which is involved in neuronal differentiation, migration and organization has also been found to be altered in MCDs [107, 108]. BCs have been reported to exhibit decreased cytoplasmic Notch-1 and reduced nuclear β-catenin expression [108]. Since the Wnt/Notch pathway influences cell size, cell cycle and cell fate, the abnormalities in Wnt/Notch signaling in BCs may be responsible for the neuropathology of MCDs [109, 110].

In addition to the principal cytoarchitectural abnormalities, immune system activation is involved in the pathophysiology of epilepsy [22]. Pro-epileptogenic immune system activation and inflammatory responses have been detected in both FCD IIa and IIb [22]. However, some investigators have found stronger expression of components of innate immunity, adaptive immunity and cytokine production in FCD IIb compared to FCD IIa. The hypothesis has been advanced that BCs are crucial drivers of inflammation in FCD IIb, with the underlying mechanism possibly stemming from a high mutational burden and consequent intrinsic activation of the mTOR [3, 22]. For instance, the expression of HLA-II and IL-1β has been shown to be dependent on the mTOR pathway [111, 112]. Additionally, the generalized anabolic activity resulting from mTOR hyperactivity may promote the nonspecific production of immune factors [113].

Meanwhile, Yang et al. investigated the link between inflammatory responses and BCs in FCD and TSC tissues, providing evidence that supports the role of BCs in initiating inflammatory response [81, 82, 85, 114]. In their studies, he and colleagues first observed the overexpression of interleukins and their receptors (IL-6/IL-6R, IL-17/IL-17) in BCs in FCD IIb, which were co-expressed with GFAP and sometimes with NF200 [81]. They also found increased expression of toll-like receptors (TLRs) and high-mobility group box 1 (HMGB1) in FCD IIb and TSC, potentially leading to the upregulation of downstream inflammatory factors in epilepsy, including FPR2, nuclear factor-κB (NF-κB), interleukin-1β (IL-1β), and tumor necrosis factor-α (TNF-α) [115]. Specifically, TLR-2 was predominantly detected in microglia/macrophage cells and BCs, indicating that they represented a significant source of pro-inflammatory molecules [77]. Secondly, increased levels of VEGFs were found in BCs, DNs and astrocytes in FCD IIb, which could contribute to astroglial activation and associated inflammatory reactions [65, 82]. Furthermore, high levels of MMP9 were detected in BCs and reactive astrocytes, which is known to be a regulator of various physiological and pathological inflammatory processes [83]. Additionally, human leukocyte immunoglobulin-like receptor B2 (LILRB2), involved in neurite growth, synaptic plasticity, and inflammatory reactions, was strongly expressed in DNs and BCs, suggesting its potential role in pathogenesis of MCDs [84].

Researchers have identified several inflammatory markers in BCs and some small glial cells, including inducible NOS, xCT, and COX-2 [85]. Furthermore, there is higher expression of CCL2 in microglia close to BCs, suggesting intercellular reactions and an additional pro-inflammatory contributions from glial cells [22, 75]. The activation of these inflammatory signaling pathways in focal malformations of cortical development may contribute to the high epileptogenicity of developmental lesions [22]. Therefore, targeting the aberrant immunogenic process of BCs during brain development could be served as a potential therapeutic approach for MCDs [22, 81, 85].

## Conclusions

Balloon cells (BCs), with their unique morphological and physiological features, have emerged as a compelling focus in epileptogenesis research. Morphologically, BCs resemble balloons and exhibit characteristics of both neurons and glial cells, indicating a developmental aberration where precursor neurons likely misinterpret signals for growth, division, and differentiation, leading to a suspended cell cycle and an undifferentiated state. Their predominant presence at the gray-white matter interface or within white matter, with limited migration to superficial layers, suggests a possible structural maladaptation. Physiologically, the lack of action potential generation and minimal response to electrical stimuli by BCs hint at their potential role in mitigating aberrant excitatory signals during epileptic episodes. The profusion of channels, receptors, and transporters on BCs, along with inhibitory fiber envelopment and demonstrated glutamate reuptake potential, indicates they may play a constructive role in regulating excitatory neurotransmission. Inflammatorily, BCs can provoke inflammation, potentially harming patients, yet also delineating the lesion area as a specific target for pharmacological intervention, possibly yielding therapeutic benefits. On a molecular level, BCs frequently correlate with aberrant activation of the mTOR pathway, leading to a spectrum of molecular and cellular distrubances, primarily observed in pathological entities, including BCs and DNs. In summary, BCs challenge the simplistic binary of being solely harmful or beneficial; they manifest a complex dualism that warrants attention.

**Fig. 4:**
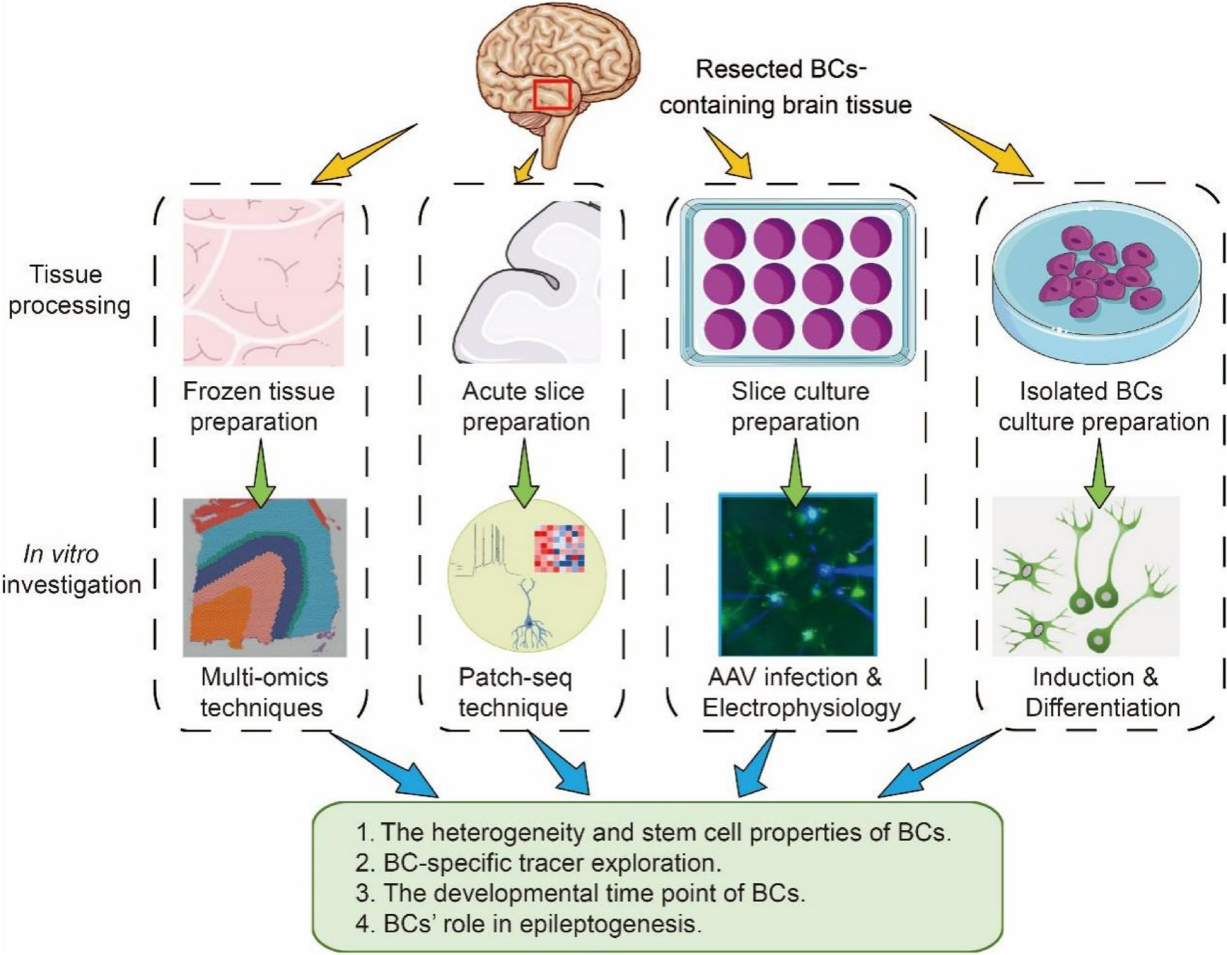
Future perspectives of BCs-containing MCDs study. Future studies on balloon cells (BCs) can be approached from multiple perspectives. Firstly, surgically resected fresh BCs-containing brain tissue can be rapidly frozen and analyzed using single-cell/spatial transcriptional and metabolomic techniques. Secondly, this fresh brain tissue can be used to prepare acute slices suitable for patch-seq analysis, enabling the integration of electrophysiological, morphological, and single-cell transcriptional data in BCs. Additionally, given the time-sensitive nature of acute slice experiments, further slices containing BCs can be cultured in an incubator for extended periods, allowing for adeno-associated virus (AAV) infection and other molecular manipulations. Finally, BCs isolated from fresh brain tissue can be cultured to study their differentiation induction and the explore potential therapeutic interventions. These methodologies aim to address critical yet unresolved questions regarding the heterogeneity and stem cell-like properties of BCs, their origins, and their role in the development of epilepsy

Despite progress in imaging, cellular electrophysiology and molecular biology, there are still essential questions that remian unanswered, necessitating further exploration. Due to technological limitations and limited availability of samples, the scientific community should exploit technological progress and innovation to enhance our comprehension of the inherent characteristics of BCs. This effort has the potential to establish more effective, less invasive, or non-invasive treatment approaches (Fig. 4).

Currently, the mechanisms underlying the divergent histopathologies resulting from mutations in the same gene or protein domain, such as the formation of BC in certain individuals with *MTOR* or *DEPDC5* mutations but not others, remain unclear. Despite numerous studies manipulating mTOR-related genes to emulate gain or loss of function, the induction of BCs in vitro or in animal models has not yet achieved [10]. Future research efforts should focus on identifying the developmental timing and the specific progenitor cell types implicated in BC genesis. In this context, a promising avenue is the instant freezing surgically resected brain tissue rich in BCs, followed by subsequent single cell/spatial transcriptional and metabolomic analysis. This approach was effectively demonstrated in Baldassari et al.’s study, where laser capture microdissection (LCM) was utilized [3].

The observed heterogeneity of BCs across various studies reaffirms their complexity, adding to the investigative challenge. A promising approach is to amalgamate multidimensional data, including electrophysiology, morphology, and single-cell transcriptomics. Furthermore, extending the viability of acute slices in culture could facilitate AAV infection and further molecular interventions. The investigation of the stem cell-like properties of BCs, particularly those that can be cultured in vitro from FCD IIb surgical specimens, offers valuable insights into their molecular dynamics, signaling pathways, and differentiation potential. Another potential direction worth exploring is the use of BC-specific tracers, which could have immediate clinical applications in pinpointing epileptic foci and guiding targeted therapy.

The development of advanced methodologies, such as sophisticated in vitro models, organoids, and humanized mouse models, holds great promise in elucidating the mechanisms underlying BC genesis and progression. These approaches have the potential to enhance our understanding of the specific etiology and pathophysiology of MCDs involving BCs.

## Data Availability

Availability of data and materials is not applicable in this study.

## Abbreviations

BCs: Balloon cells
CNS: Central nervous system
DNs: Dysmorphic neurons
FCD: Focal cortical dysplasia
GJs: Gap junctions
HME: Heminegaloencephaly
LCM: Laser-capture microdissection
MCDs: Malformations of cortical developments
mTOR: Mammalian target of rapamycin
PCWs: Postconceptional weeks
PV: Parvalbumin
Rb: Retinoblastoma protein
RG: Radial glia
R-point: Restriction point
TSC: Tuberous sclerosis complex
VZ: Ventricular zone
